# Lactic acid bacteria, potentially probiotic, in whole-grain pet biscuits: survival and technological potential of millet-based formulations (*Pennisetum glaucum*)

**DOI:** 10.1007/s42770-026-02091-8

**Published:** 2026-09-10

**Authors:** Wendy Carla Dantas Rocha, Isabel Luísa Ribeiro de Abreu Teixeira, Maria Eduarda Costa Campos, Taynan Jonatha Neves Costa, Isabella Maciel Costa, Debora Cristina Sampaio de Assis, Felipe Machado Trombete, Washington Azevedo da Silva, Erika Ramos de Alvarenga, Fabiola de Oliveira Paes Leme, Verônica Ortiz Alvarenga, Bruna Maria Salotti de Souza

**Affiliations:** 1 Department of Technology and Inspection of Products of Animal Origin, School of Veterinary, Federal University of Minas Gerais, Belo Horizonte, 31270-901 Minas Gerais Brazil; 2 Department of Food, Faculty of Pharmacy, Federal University of Minas Gerais, Belo Horizonte, 31270-901 Minas Gerais Brazil; 3 Food Engineering, Federal University of São Joao del Rei, Sete Lagoas, 35702-031 Minas Gerais Brazil; 4 Department of Animal Science, School of Veterinary, Federal University of Minas Gerais, Belo Horizonte, 31270-901 Brazil; 5 Department of Veterinary Clinical and Surgical Medicine, School of Veterinary, Federal University of Minas Gerais, Belo Horizonte, 31270-901 Minas Gerais Brazil; 6 Food Microbiology Laboratory, School of Veterinary, Federal University of Minas Gerais, Belo Horizonte, Brazil

**Keywords:** Canine, Functional, Millet, Wheat

## Abstract

The relationship between humans and pets has driven the development of functional foods. This study aimed to develop pet biscuits containing *Lacticaseibacillus paracasei* GV17 and *Lactococcus lactis* GV103 and to evaluate the effects of wheat flour, millet flour, and a combination of both on the stability of lactic acid bacteria (LAB), resistance to simulated gastrointestinal tract (GIT) conditions, technological characteristics, and viability during storage. Three formulations were prepared using wheat flour (WF), millet flour (MF), and a mixture of flours (WMF), in which the GV17 and GV103 strains, as well as their combined culture (GV17.103), were incorporated into the biscuit cavities. The type of flour significantly influenced the physicochemical and technological characteristics of the formulations. A higher luminosity value was observed in the WF formulation (76.31), while the WMF showed greater hardness (46.18 N), indicating greater resistance to breakage. The MF presented higher lipid (24.80%) and fiber (5.76%) contents, WMF higher moisture (12.49%), and WF higher carbohydrate (55.95%). All formulations recover LAB counts to values above 7.0 log CFU/mL at the end of simulated digestion. WF103, MF103, and MF17.103 stood out for their storage stability. These results demonstrate that the tested matrices are suitable for the development of pet biscuits containing potentially probiotic LAB strains.

**Supplementary Information:**

The online version contains supplementary material available at https://doi.org/10.1007/s42770-026-02091-8.

## Introduction

Contemporary demographic changes have led to smaller family sizes and increased pet ownership [1]. Higher rates of pet ownership are positively correlated with the growing demand for nutritionally enhanced pet foods [2]. Furthermore, concerns have been raised regarding the indiscriminate use of antibiotics in dogs, as this practice may contribute to the emergence of multidrug-resistant bacteria [3].

In response to current market demands, pet food formulations have increasingly incorporated ingredients with well-documented health benefits, aligning with pet owner preferences [4]. A promising strategy for disease prevention and the maintenance of intestinal eubiosis is the administration of probiotics [5]. In terms of gene content and dietary responsiveness, the intestinal microbiota of dogs shows notable similarities to that of humans [6]. Therefore, probiotic supplementation may help modulate the intestinal microbiota, improving the quality of life of both animals and their owners who share the same environment and activities [7].

By definition, probiotics are live microorganisms that, when administered in adequate amounts, confer health benefits on the host [8]. According to the literature, autochthonous isolates of lactic acid bacteria (LAB) represent a natural source of promising candidates for use as starter cultures, adjuncts, or probiotics [9].

In addition to considering the physiological effects of LAB on the host, it is essential to evaluate the stability and viability of probiotics throughout the shelf life of the product in which they are incorporated. The stability of LAB within the food matrix depends on factors such as temperature, humidity, and oxygen exposure, as well as on the potential for delivery within a prebiotic matrix [10].

Several studies have sought to optimize the use of different grains in small animal feeds to improve the nutritional and technological quality of rations. Millet (*Pennisetum glaucum*) stands out as a grain with high nutritional value, notable fiber and mineral contents, and a rich profile of phenolic compounds. In addition, millet has a lower glycemic index than several refined cereals and presents higher levels of these components compared with wheat [11].

Despite its recognized nutritional benefits, wide availability, and low cost, millet remains underutilized in Brazil, particularly in animal feed. The incorporation of millet flour combined with potentially probiotic LAB into dog biscuit formulations represents a promising strategy for delivering functional compounds capable of promoting canine health. In this context, the present study aimed to develop functional dog biscuits formulated with wheat flour, millet flour, and their combination, containing the potentially probiotic strains *Lacticaseibacillus paracasei* GV17 and *Lactococcus lactis* GV103, applied individually or in combination. Additionally, this study sought to identify the formulation that best preserves LAB viability while exhibiting adequate technological performance. With the hypothesis that millet-based formulations would provide greater protection of LAB viability during storage and simulated gastrointestinal exposure than wheat-based formulations.

## Materials and methods

### Lactic acid bacteria

Two potentially probiotic LAB strains from the Culture Collection of the Department of Technology and Inspection of Products of Animal Origin, Federal University of Minas Gerais (DTIPOA/UFMG, Belo Horizonte, MG, Brazil), were used in this study. *Lacticaseibacillus paracasei* GV17 and *Lactococcus lactis* GV103 were previously evaluated and classified as potentially probiotic [12]. Although *L. lactis* is not considered a dominant or stable member of the canine gut microbiota, probiotic effects do not necessarily require permanent intestinal colonization and may result from transient interactions with the host and resident microbiota. Nevertheless, the ecological compatibility and persistence of this strain in the canine gastrointestinal tract remain to be established through in vivo studies.

The strains were previously isolated from Minas artisanal cheese, produced in the Campos das Vertentes region of Minas Gerais [13]. The LAB strains were identified by matrix-assisted laser desorption/ionization time-of-flight mass spectrometry (MALDI-TOF MS) using the Microflex™ system (Bruker Daltonics, Bremen, Germany) and the manufacturer’s database (Library BDAL V13.0.0.2). Identification criteria followed the manufacturer’s recommendations: scores ≥ 2.000 indicated species-level identification; scores ≥ 1.700 and < 2.000 indicated genus-level identification; and scores < 1.700 were considered unreliable for identification. Both LABs presented scores above 2.000.

LAB strains were cultured in De Man, Rogosa, and Sharpe (MRS) broth, dehydrated powder carrier (AS-6429, Biolog, Hayward, CA, USA) and stored at − 80 °C in the presence of 20% (v/v) sterile glycerol as a cryoprotectant. For reactivation, isolated LAB strains were inoculated (2% v/v) into 10 mL of MRS broth and incubated aerobically at 37 °C for 48 h [14].

The research complies with the regulations of the National System for the Management of Genetic Heritage and Associated Traditional Knowledge (SisGen), registered under number A14B3CC.

### Development of dog biscuits

For the dough base, three dog biscuit formulations were prepared according to González-Forte et al. [15], with minor modifications. Briefly, all formulations contained 2.5 g of NaCl, 18.5 g of sucrose, 2.0 g of sodium bicarbonate, 4.5 g of monobasic sodium phosphate, 24.75 g of sunflower oil, and 60 mL of distilled water. The formulations were then divided and supplemented with 123.75 g of type 1 wheat flour (WF), 123.75 g of ground millet (*Pennisetum glaucum*) ADR9070 flour (MF), or a combination of 61.87 g of type 1 wheat flour and 61.87 g of ground millet ADR9070 flour (WMF).

The ADR9070 millet grains were previously processed using an industrial blender (Siemsen^®^, model LS-04) under standardized processing conditions to obtain the flour. The resulting flour was sieved through a 500-µm mesh (Bertel Industria Metallurgical Ltda., Caieiras, SP, Brazil) to standardize particle size and remove larger particles before use in biscuit preparation. The selected particle size was established as part of the experimental protocol for biscuit formulation; optimization of flour particle size was not within the scope of this study. When supplemented with LAB, the biscuit formulations were classified as described in Table 1 and produced in triplicate.

**Table 1:** Biscuit formulations intended for feeding dogs, enriched with flour and lactic acid bacteria, which are potentially probiotic

Identification	Flour	Lactic acid bacteria
WF17	Wheat flour type 1	*Lbs. paracasei* GV17
MF17	Millet flour ADR9070	
WMF17	Wheat flour and millet	
WF103	Wheat flour type 1	*L. lactis* GV103
MF103	Millet flour ADR9070	
WMF103	Wheat flour and millet	
WF17.103	Wheat flour type 1	Association of *Lbs. paracasei* GV17 e *L. lactis* GV103
MF17.103	Millet flour ADR9070	
WMF17.103	Wheat flour and millet	

#### Preparation of dog biscuits

The dry ingredients used in biscuit preparation were weighed separately on a precision scale (BEL Engineering, model M503, Italy) and thoroughly mixed. Subsequently, oil and water were added. The ingredients were manually mixed according to the formulation until a homogeneous and cohesive dough was obtained. The dough was then rolled to a thickness of 0.8 cm to ensure even cooking and shaped using a circular cutter with a 3.0 cm diameter. Measurements were taken with a digital caliper (Mitutoyo Worldwide, São Paulo, Brazil).

The biscuits were placed on a baking sheet, and a small cavity was created in the center of each biscuit using a glass rod to allow for the subsequent addition of the LAB suspension after baking. For industrial-scale production, alternative incorporation strategies, including spray incorporation into the pulp, can be beneficial. Baking was performed in a preheated industrial electric oven (MAKEL Indústria Metalúrgica Ltda., São Paulo, Brazil) at 170 °C. The biscuits were baked for 15–20 min. The cooking parameters were chosen to reflect standard biscuit production.

##### Addition of potentially probiotic LAB

After an incubation period of 48 h, aliquots of the bacterial suspensions (1.0 mL), according to each formulation, were centrifuged at 10.000 × g for 10 min at 25 °C, and the supernatants were discarded. The resulting pellets were resuspended in 0.1 mL of phosphate-buffered saline (PBS) and gently mixed. For formulations containing both GV17 and GV103, 0.05 mL of each LAB suspension was added to the biscuits, 6.0 to 8.0 log CFU/mL was added to the biscuits. Under sterile conditions, the entire bacterial suspension was deposited into the depression previously formed on the biscuit surface [15].

##### Coating with cornstarch

A gelatinized cornstarch suspension was applied to the biscuits using a sterile brush, in order to create a protective barrier. The suspension (30 g/L cornstarch) was gelatinized in a Thermomix (Cloyes-sur-le-Loir, France) at 90 °C for 30 min. After cooling to room temperature, glycerol was added at a concentration of 0.9 mL per liter of suspension. Following coating, the biscuits were dried at room temperature under sterile conditions and subsequently stored at 4 °C in sterile packaging [15]. Refrigeration was intentionally selected as an experimental strategy to maximize the stability of viable LAB cells during storage. Storage temperature is an important determinant of probiotic stability, and lower temperatures can help minimize viability losses during storage.

### Technological characterization of biscuits

#### Color evaluation

Objective color evaluation was performed on the surface of the biscuits. Instrumental color parameters (L*, a*, and b*) were measured using a colorimeter (CM-2300d spectrophotometer; Konica Minolta). Measurements were obtained from 12 biscuit samples representing the three flour formulations, analyzed in triplicate, and the results were expressed as the mean of the readings.

#### Texture profile assessment

Instrumental texture analysis was performed using a texture analyzer (TA.XTplus, Stable Micro Systems, Godalming, UK). A fracture test was conducted under the following conditions: pre- and post-test speeds of 1 mm/s, a test speed of 0.5 mm/s, and a maximum compression of 60%, using a 5 mm compression probe. Twelve samples per formulation were evaluated. From the force–strain curves, the maximum fracture force and the slope at the point of maximum force were determined [16].

#### Proximate composition

Analyses of moisture, crude protein, lipids, crude fiber, ash, and chlorides were performed. Moisture content was determined by oven drying at 105 °C until constant weight [17]. Crude protein was quantified using the Kjeldahl method, involving distillation and titration with 0.1 mol/L HCl, and the nitrogen content was converted to protein using a factor of 6.25 [17]. Lipids were extracted by Soxhlet extraction using petroleum ether [17], and ash content was determined by incineration in a muffle furnace at 550 °C for 5 h [17].

Crude fiber was determined after sequential acid and alkaline digestion, followed by filtration, drying, and incineration in a muffle furnace for 4 h [17]. Chloride content was determined by titration of the water-soluble mineral residue with 0.1 N silver nitrate, using potassium chromate as an indicator [17]. The nitrogen-free extract, used as an estimate of carbohydrate content, was calculated as the difference between dry matter and the sum of crude protein, lipids, crude fiber, and ash.

### Microbiological analysis of biscuits

#### LAB viability during storage

The viability of LAB was evaluated on days 1, 7, 14, 21, and 28 after production. Biscuits supplemented with *Lbs. paracasei* GV17, *L. lactis* GV103, and their combinations across the different formulations were stored in sterile packaging under refrigeration at 4 °C for 28 days.

Samples (10 g) were weighed into sterile containers, mixed with 90 mL of 0.85% saline solution, and shaken for 1 min in a stomacher (NT120 Novatécnica^®^, São Paulo, Brazil). Serial dilutions were prepared, and eight dilutions were surface plated onto MRS agar in triplicate. Plates were incubated aerobically at 37 °C for 48 h. Viability was expressed as log CFU/g and represented as survival potential (δ), calculated as the difference between final counts and initial counts at the beginning of the storage period. Negative δ values ≤ − 0.5 log indicated that the formulation did not favor LAB survival [18].

#### Viability of LAB under simulated gastrointestinal tract conditions (GIT)

The simulated gastrointestinal assay was performed as an in vitro screening approach to evaluate the tolerance and survival of the potentially probiotic LAB under sequential GIT conditions. The procedure was adapted from the method described by Bautista-Gallego et al. [19], which was originally developed for the simulation of human GIT digestion. The assay was not intended to reproduce the canine gastrointestinal tract in its entirety, but rather to expose the bacterial cells to the major physicochemical stresses associated with gastrointestinal transit, including acidic gastric conditions and subsequent exposure to bile salts and pancreatic enzymes. Therefore, the results were interpreted as an indication of relative gastrointestinal tolerance rather than as a direct prediction of in vivo survival in dogs.

Simulated gastric juice was prepared in a buffered solution. The pH was adjusted to 2.0 using 1 M HCl (Synth, Diadema, SP, Brazil). Simulated enteric juice was prepared using disodium hydrogen phosphate heptahydrate (Na_2_HPO_4_ 50.81 g/L) (Synth, Diadema, SP, Brazil) and NaCl (8.5 g/L). The pH was adjusted to 8.0 using anhydrous KH_2_PO_4_ + K₂HPO₄. Biscuit samples (0.5 g) were crushed, and 1.0 mL of phosphate-buffered saline (PBS) was added, followed by centrifugation at 10,000 × g for 10 min at 4 °C. The pellets were then washed with PBS (0.1 M, pH 7.2), and the cells were resuspended in simulated gastric juice to obtain concentrations of 8 to 9 log CFU/mL. Prior to use, porcine gastric mucosal pepsin (P7000; 3 g/L; Sigma-Aldrich) was added, and the suspensions were incubated at 37 °C for 120 min in a water bath.

LAB cells collected during the gastric digestion phase were serially diluted in phosphate-buffered saline (PBS), and viable counts were determined by surface plating on agar at time zero (T0) and after 120 min (T120) of incubation. To simulate small intestinal conditions, simulated enteric juice was used. Prior to use, porcine pancreatin (P3292; 1 g/L; Sigma-Aldrich) and bile salts (B8631; 3 g/L; Sigma-Aldrich) were added. LAB cells recovered from the gastric phase were washed with PBS, resuspended, and subsequently incubated for 360 min at 37 °C in a water bath. Viable counts were then determined as previously described (T360). All results were expressed as log CFU/mL. All enzyme solutions were freshly prepared and sterilized by filtration through a 0.22 μm membrane filter, PES low-protein-binding (Merck Millipore Ltd., Cork, Ireland) prior to use.

### Statistical analysis

All experiments were conducted using three independent biscuit productions performed on different days (*n* = 3). For each production, the analyses were performed in triplicate. The triplicate measurements within each production were considered analytical replicates and were not treated as independent biological replicates in the statistical analysis.

The statistical analysis of the technological characterization of the biscuit was performed using the InfoStat statistical software version 2020. The results were subjected to analysis of variance (ANOVA) and evaluated using Tukey’s statistical tests, at a 5% significance level (*p* < 0.05). To evaluate the bacteria count, linear regression models were fitted considering time (days), bacteria lineage (GV17, GV103 and GV17-103) and formulation (MF, WF and WMF). Bacteria lineage and formulation were coded as indicator variables (binary variables). The GV103 and MF were the reference groups for bacteria lineage and formulation, respectively. Statistical analyses were performed using R software [20] and Tidyverse package [21] was used for data wrangling (dplyr) and data visualization (ggplot2).

## Results

### Technological characterization of biscuits: evaluation of color, texture, and proximate composition

In dog biscuits, the highest L* value was observed in the formulation made with wheat flour (WF 76.31), followed by the wheat–millet blend (WMF 59.68) and the millet flour (MF 43.19) formulations (Table 2). The MF formulation exhibited higher a* values, associated with redness, and higher b* values, associated with yellowness, compared with the WF and WMF formulations (*p* < 0.05) (Table 2).

**Table 2:** Effect of adding wheat flour (WF), millet flour (MF), and a combination of wheat and millet flour (WMF) on the color parameters of dog biscuits

Formulation	L*	a*	b*
**WF**	76.31 ± 5.63^a^	12.04 ± 1.47^b^	47.97 ± 4.56^c^
**MF**	43.19 ± 1.17^c^	15.63 ± 0.65^a^	60.25 ± 1.79^a^
**WMF**	59.68 ± 6.29^b^	12.86 ± 1.84^b^	51.75 ± 3.18^b^

Different lowercase letters in the column indicate a significant difference between the formulations according to Tukey’s test (*p* < 0.05). Results are expressed as mean ± standard deviation (*n* = 12)

The highest hardness value was observed in the WMF formulation (46.18 N), indicating greater resistance to breakage (Table 3). In contrast, the WF (26.65 N) and MF (23.17 N) formulations exhibited hardness values approximately 20 N lower than that of the WMF formulation (*p* < 0.05).

**Table 3:** Effect of adding wheat flour (WF), millet flour (MF), and a combination of wheat and millet flour (WMF) on the hardness parameter of dog biscuits

Formulation	Hardness (*N*)
**WF**	26.65 ± 5.89^b^
**MF**	23.17 ± 4.84^b^
**WMF**	46.18 ± 12.51^a^

Different lowercase letters indicate a significant difference between formulations according to Tukey’s test (*p* < 0.05). Results are expressed as mean ± standard deviation (*n* = 12)

The moisture content of the biscuits ranged from 9.48% in the WF formulation to 12.49% in the WMF formulation (Table 4), with no significant difference between the WF and MF formulations (*p* > 0.05). The MF (5.67%) and WMF (5.33%) formulations exhibited the highest ash contents (Table 4). Millet is recognized as a rich source of dietary fiber and resistant starch and contains high levels of minerals and trace elements, particularly potassium, calcium, phosphorus, and iron, compared with other cereals such as wheat [22]. This nutritional profile likely contributed to the higher ash content observed in the MF and WMF formulations, as well as to the higher chloride titration value (1.63%) recorded for the MF formulation compared with the WF and WMF formulations (*p* < 0.05) (Table 4).

**Table 4:** Proximate composition of biscuits intended for feeding dogs, made with wheat flour (WF), millet flour (MF) and a combination of wheat flour and millet (WMF)

Formulation	Moisture (%)	Ash (%)	Chlorides (%)	Proteins (%)	Lipids (%)	Fiber (%)	Carbohydrates (%)
**WF**	9.48 ± 2.16^b^	4.67 ± 0.95^b^	1.43 ± 0.19^b^	12.36 ± 1.02^a^	17.77 ± 1.88^b^	0.95 ± 0.11^c^	55.95 ± 2.71^a^
**MF**	9.75 ± 1.59^b^	5.67 ± 0.50^a^	1.63 ± 0.13^a^	13.17 ± 0.58^a^	24.80 ± 3.40^a^	5.76 ± 1.15^a^	40.23 ± 4.32^b^
**WMF**	12.49 ± 2.01^a^	5.33 ± 1.96^ab^	1.48 ± 0.23^b^	12.91 ± 1.52^a^	22.65 ± 2.08^a^	3.45 ± 0.52^b^	42.98 ± 3.58^b^

Different lowercase letters in the column indicate a significant difference between the formulations according to Tukey’s test (*p* < 0.05). Results are expressed as mean ± standard deviation (*n* = 18)

The protein content did not differ among the three biscuit formulations (*p* > 0.05), ranging from 12.36% in the WF formulation to 12.91% in the WMF formulation and 13.37% in the MF formulation. Regarding lipid content, the MF formulation exhibited a 7.03% higher value than the WF formulation (*p* < 0.05) (Table 4). The fiber content of the WF, MF, and WMF formulations was 0.95%, 5.65%, and 3.45%, respectively. The WF formulation showed the highest carbohydrate content (55.95%), significantly differing from the MF (40.23%) and WMF (42.98%) formulations (*p* < 0.05) (Table 4). This finding can be attributed to the relatively higher levels of fiber, lipids, and minerals in millet, which reduce the proportion of starch in the final formulation [23].

### Microbiological analysis of biscuits: LAB viability during storage and under simulated GIT conditions

**Fig. 1:**
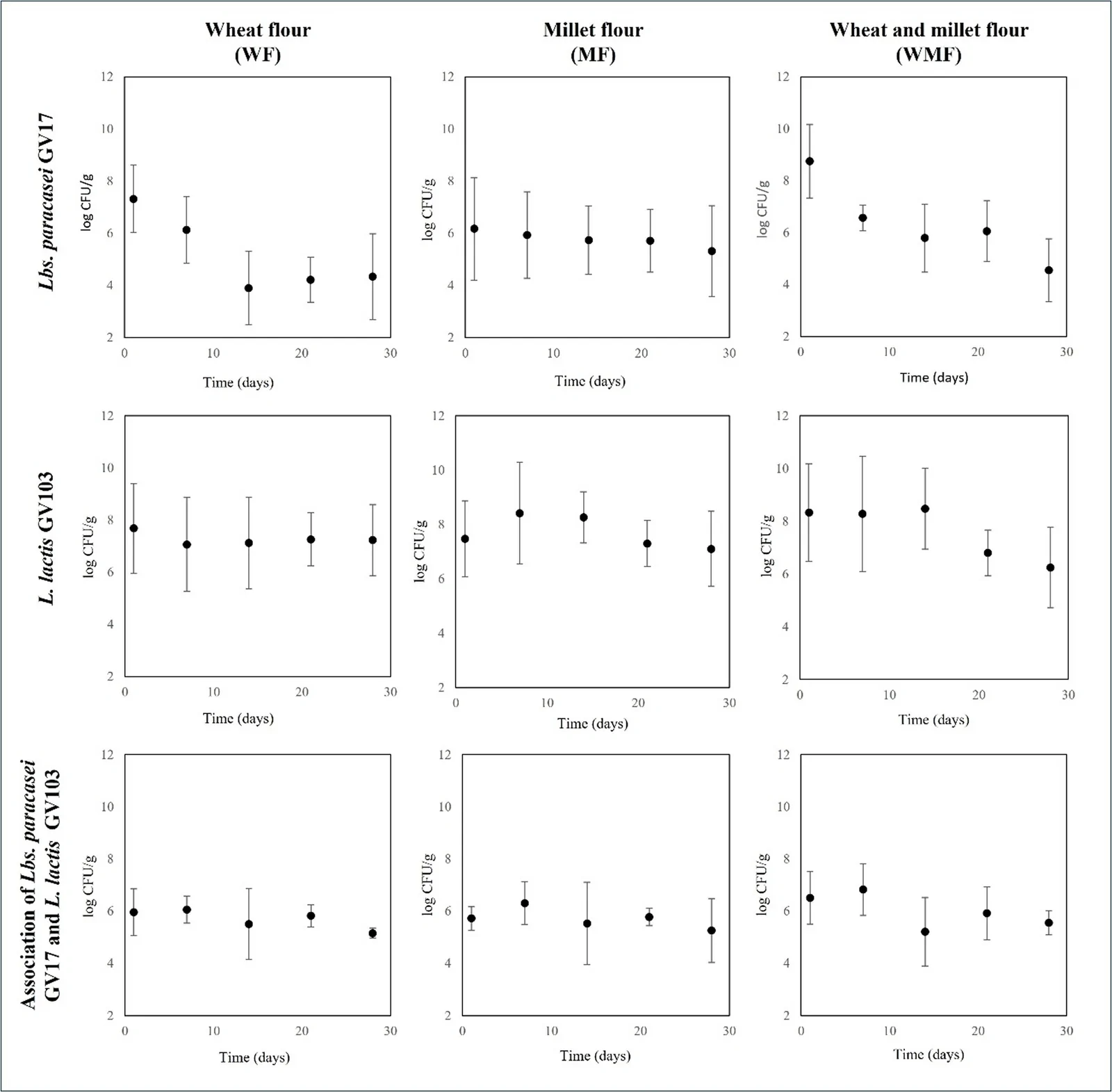
Viability (log CFU/g) of lactic acid bacteria added to biscuit formulations intended for dog food, throughout shelf life. Whole-grain pet biscuits made with millet flour (*Pennisetum glaucum*) and probiotic lactic acid bacteria

The behavior of the evaluated LAB strains, whether applied individually or in consortium, varied across the different formulations over the 28 days of refrigerated storage (Fig. 1). Specifically, the WF17 formulation exhibited a reduction of 3.43 log CFU/g in LAB counts, whereas the WMF17 formulation showed a greater reduction of 4.20 log CFU/g.

Biscuits supplemented with GV17 showed LAB counts of 4.34 log CFU/g (WF17), 4.55 log CFU/g (WMF17), and 5.32 log CFU/g (MF17) after 28 days of storage. Biscuits supplemented with the combined culture of GV17 and GV103 exhibited counts ranging from 5.15 to 5.55 log CFU/g, corresponding to the WF17.103 (5.15 log CFU/g), MF17.103 (5.26 log CFU/g), and WMF17.103 (5.55 log CFU/g) formulations. The highest LAB counts at the end of the 28-day shelf life were observed in biscuits formulated with wheat flour, millet flour, or their combination when supplemented with GV103, reaching 6.24 log CFU/g in WMF103, 7.10 log CFU/g in MF103, and 7.23 log CFU/g (WF103).

**Fig. 2:**
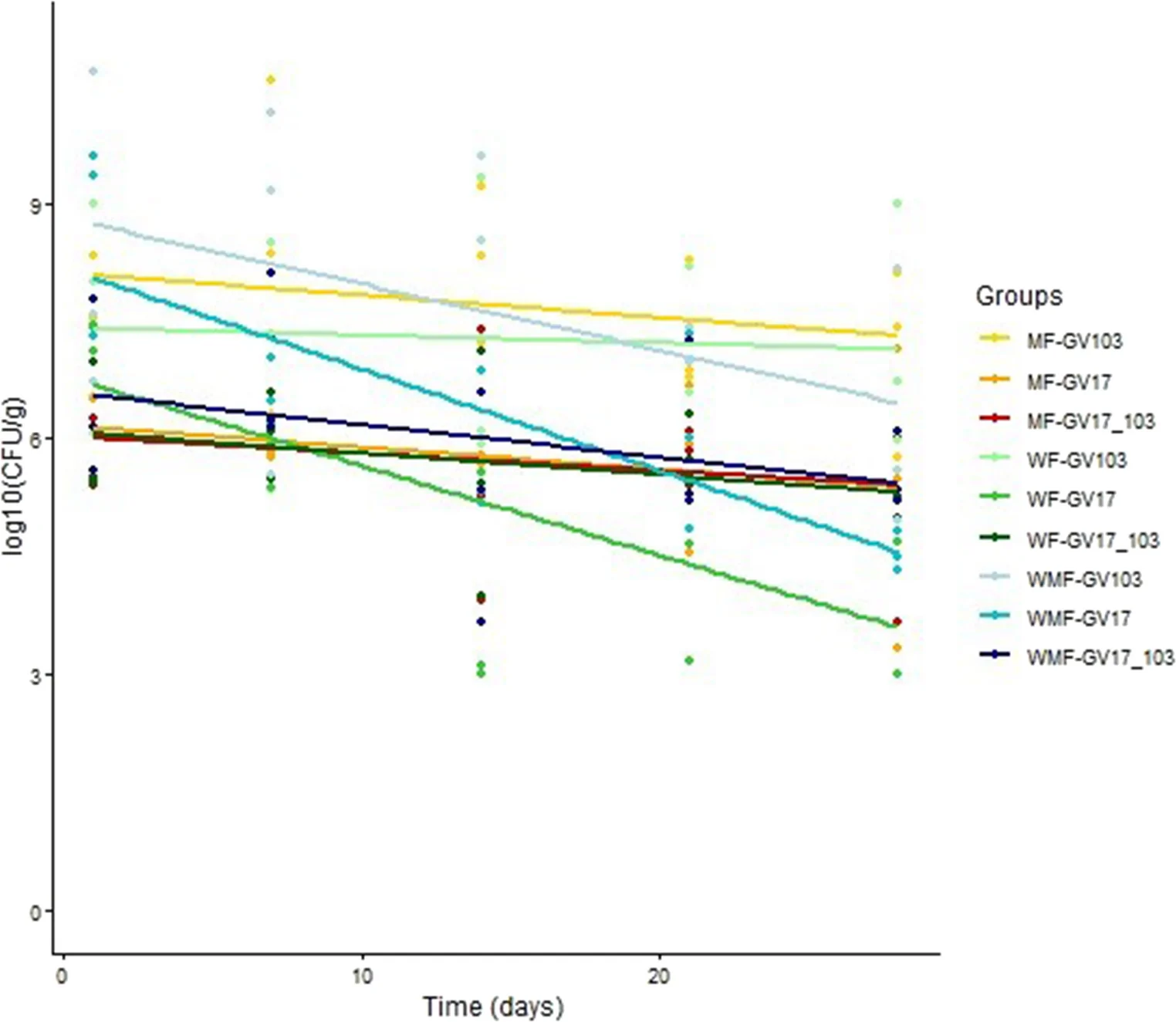
Fig 2. Regression model of log10(bacteria count) over time with different formulation (R2 = 0.42). WF17: biscuit made with wheat flour and *Lbs. paracasei* GV17; MF17: biscuit made with millet flour and *Lbs. paracasei* GV17; WMF17: biscuit made with wheat flour and millet with added *Lbs. paracasei* GV17; WF103: biscuit made with wheat flour and *L. lactis* GV103; MF103: biscuit made with millet flour and *L. lactis* GV103; WMF103: biscuit made with wheat flour and millet with added *L. lactis* GV103; WF17.103: biscuit made with wheat flour and *Lbs. paracasei* GV17 combined with *L. lactis* GV103; MF17.103: biscuit made with millet flour and *Lbs. paracasei* GV17 combined with *L. lactis* GV103; WMF17.103: biscuit made with wheat flour and millet with added *Lbs. paracasei* GV17 associated with *L. lactis* GV103

The bacterial count of GV17 and GV17 and 103 consortium was lower than that of GV103 (Supplementary material 2). The bacterial count in WMF was significantly higher than the bacterial count in MF (Fig. 2). A significant interaction was found between WMF and time in the bacterial count. The coefficient of determination (R^2^) of the model was 0.451, the adjusted R^2^ was 0.42 and maximum R2 was 0.77. Although the variability within the treatment represented an important source of variation in the present study, the model explained up to 77% of the variability, accounting for the variability inherent in the triplicates.

When the survival potential (δ) of LAB was evaluated across the different formulations, the WF103 (δ = −0.45 log), MF103 (δ = −0.37 log), and MF17.103 (δ = −0.46 log) exhibited δ values above the − 0.5 log threshold, indicating that these samples were the most effective at preserving LAB viability over 28 days of refrigerated storage. Overall, formulations containing the GV103 strain, particularly those incorporating millet flour (MF), demonstrated superior LAB survival performance.

The MF17 (10.16 log CFU/mL), WF103 (9.30 log CFU/mL), and MF103 (10.20 log CFU/mL) formulations maintained the highest LAB counts after passage through the gastric phase, whereas the remaining formulations exhibited counts below 7.53 log CFU/mL (Table 5). No significant differences (*p* > 0.05) in LAB counts were observed throughout the simulated gastrointestinal tract evaluation for the MF17, WMF17, WF103, and WMF103 formulations. In contrast, the MF17.103 formulation (*p* < 0.05) showed a reduction of 2.53 log CFU/mL from the initial time point to the gastric phase, followed by an increase of 0.62 log CFU/mL during the intestinal phase (Table 5).

**Table 5:** Viability (log CFU/mL) of lactic acid bacteria added to biscuit formulations intended for feeding dogs, after exposure to simulated GIT conditions

Formulation	T0	Gastric phase	Enteric phase
		T120	T360
**WF17**	8.31 ± 0.^41abcB^	7.53 ± 0.80^bcdB^	10.07 ± 0.21^aA^
**MF17**	9.87 ± 1.02^aA^	10.16 ± 0.28^abA^	9.18 ± 1.02^aA^
**WMF17**	7.90 ± 1.01^abcdA^	5.77 ± 2.40^dA^	9.80 ± 2.23^aA^
**WF103**	7.49 ± 1.33^bcdA^	9.30 ± 0.89^abcA^	7.67 ± 0.93^aA^
**MF103**	8.36 ± 0.61^abcB^	10.20 ± 0.35^aA^	9.10 ± 1.01^aAB^
**WMF103**	9.26 ± 0.94^abA^	7.49 ± 0.50^cdA^	9.35 ± 1.17^aA^
**WF17.103**	6.59 ± 0.11^cdB^	5.49 ± 0.20^dC^	8.60 ± 0.30^aA^
**MF17.103**	9.20 ± 0.17^abA^	6.67 ± 0.06^cdC^	8.46 ± 0.28^aB^
**WMF17.103**	6.10 ± 0.17^dB^	6.58 ± 0.27^dB^	7.20 ± 0.17^aA^

Different lowercase letters in the same column indicate a significant difference (*p* < 0.05) between the formulation according to Tukey test. Different uppercase letters in the same row indicate a significant difference (*p* < 0.05) between the times evaluated for the same formulation according to Duncan test. Results are expressed as mean ± standard deviation (*n* = 9). WF17: biscuit made with wheat flour and *Lbs. paracasei* GV17; MF17: biscuit made with millet flour and *Lbs*. *paracasei* GV17; WMF17: biscuit made with wheat flour and millet with added *Lbs. paracasei* GV17; WF103: biscuit made with wheat flour and *L. lactis* GV103; MF103: biscuit made with millet flour and *L. lactis* GV103; WMF103: biscuit made with wheat flour and millet with added *L. lactis* GV103; WF17.103: biscuit made with wheat flour and *Lbs. paracasei* GV17 combined with *L. lactis* GV103; MF17.103: biscuit made with millet flour and *Lbs. paracasei* GV17 combined with *L. lactis* GV103; WMF17.103: biscuit made with wheat flour and millet with added *Lbs. paracasei* GV17 associated with *L. lactis* GV103

The WF103 (7.67 log CFU/mL) and WMF17.103 (7.20 log CFU/mL) biscuits exhibited the lowest LAB counts after 360 min of exposure to simulated gastrointestinal tract (GIT) conditions. In contrast, the MF103 (9.10 log CFU/mL) and MF17.103 (8.46 log CFU/mL) formulations showed high survival rates throughout the 28-day storage period and maintained good viability under simulated GIT conditions, indicating that the developed matrices provide a favorable environment for LAB survival and the expression of their probiotic potential.

## Discussion

Color is an important quality attribute in foods and is often influenced by cooking reactions and the characteristics of the raw materials used [24]. The color differences observed among the WF, MF, and WMF formulations may be associated with the naturally darker color of millet and its higher contents of phenolic compounds and minerals (Supplementary material 1). In addition, Arepally et al. [25] reported that the darker color of millet-based products can be attributed, among other factors, to their higher mineral content, which is consistent with the appearance observed in the MF biscuit (Table 4).

In animal feed, color is a quality attribute directly associated with acceptance by pet owners [26]. The relationship between sensory perception and emotion in humans may suggest that cognitive and affective processes are shaped by past perceptual experiences, which, in turn, influence product choice [27].

Another relevant sensory attribute of dog food is texture. A study by Valencia et al. [1] reported that preliminary sensory analyses of carrot-based gummy prototypes indicated that dogs did not prefer products with higher levels of firmness. The authors suggested that this lack of preference may be related to dogs associating the product with a toy rather than with an edible treat.

The higher hardness observed in the WMF formulation may be associated with its relatively low fiber content [28], which was 3.45%. In the present study, the lowest fiber content was found in the WF formulation (0.95%), which presented a hardness value of 26.65 N, 3.48 N higher than the minimum value obtained. The presence or absence of a gluten network can impact the hardness of biscuits [29]. MF, characterized by gluten-free flour, was the formulation that presented the lowest hardness (23.17%).

When evaluating the proximate composition of dog biscuits, all formulations exhibited moisture contents below 14%, which may contribute to an extended shelf life by reducing susceptibility to microbial growth [30]. Conversely, such low moisture levels may create an unfavorable environment for maintaining LAB viability.

The higher ash content may promote moisture absorption in baked goods due to the elevated mineral concentration and its interaction with the food matrix. In addition, the higher ash and sodium contents characteristic of millet may be associated with the presence of the husk, which concentrates minerals and micronutrients [23]. The high mineral content of the millet pericarp and husk likely contributes to the increased ash levels observed in biscuits formulated with this cereal [31].

The lack of significant variation in protein content among the different formulations can be attributed to the similar protein levels of the raw materials used. Crude protein contents in raw millet grains and other cereals have been reported to be comparable, suggesting that minor differences among raw materials may not result in meaningful changes in food formulations [32]. Munshi and Dashora [30] reported that higher protein contents can impart softness to biscuits. However, despite their similar protein levels (Table 4), the WF, MF, and WMF formulations exhibited different hardness values when compared with the WF biscuit.

Although the millet germ represents only a small fraction of the grain, it is rich in lipids [23]. This characteristic may explain the higher fat content observed in formulations containing millet (Table 4), reflecting the intrinsic composition of this cereal, which is naturally richer in lipids than refined wheat flour.

The higher fiber content observed in millet flour can be attributed to the presence of cellulose and hemicellulose in the millet husk, which is reflected in the fiber composition of the biscuits [23]. Millet fiber comprises both soluble fractions, such as β-glucans, arabinoxylans, and pectins, and insoluble fractions, including cellulose and hemicellulose, as well as resistant starch [31].

According to Jacob et al. [33], millet contains nearly twice the fiber content of whole wheat. The lower fiber content observed in the WF formulation (Table 4) may be attributed to wheat processing. Roller milling primarily aims to produce refined flours composed predominantly of the endosperm; during this process, the outer grain layers (bran) and the germ are removed, resulting in reduced levels of fiber, minerals, vitamins, and phenolic compounds [34].

The wheat flour used in the WF formulation is predominantly composed of endosperm rich in starch, which may explain the higher carbohydrate content observed in the WF formulation (55.95%). The predominance of starch in the wheat endosperm, together with the presence of arabinoxylans and β-glucans, supports the consistency of this result with the refined nature of wheat flour [34].

Technologically, the biscuits developed with wheat flour, millet flour, and their combination exhibited characteristics consistent with the raw materials and processing conditions employed. The observed variations were mainly associated with differences in flour composition, which influenced the product structure.

The incorporation of LAB into biscuits enabled the evaluation of the solid matrix as a vehicle for potentially probiotic microorganisms. LAB survival is directly influenced by formulation composition and the conditions imposed during processing. For probiotics to exert their effects, it is essential that they withstand processing and the food storage period, making strain selection and the maintenance of cell viability key technological challenges.

The stability of the GV103 strain in the MF formulation may be associated with its ability to metabolize the starch present in the food matrix. Although this trait is uncommon, some amylolytic LAB strains harbor the *amyA* gene, which encodes the production of extracellular α-amylase. This enzyme enables the utilization of starch as a substrate for lactic acid production, which may favor the maintenance of cell viability during storage [35].

Currently, there is no universally established probiotic dose for dogs, as the effective dose depends on the strain, formulation, and target condition. The levels commonly considered adequate for probiotic foods in humans may serve as a reference point when developing probiotic products for companion animals [36]. Based on the consensus applied to humans, all formulations tested, containing LAB and different flours, maintained viable cell counts above 7.0 log CFU/mL (Table 5) after passage through the GIT [37]. A previous study evaluated the viability of the GV17 and GV103 strains, in isolation, under simulated GIT conditions [12]. There was no difference (*p* > 0.05) in counts between the gastric and intestinal phases, and the strains showed counts of 8.18 log CFU/mL (GV17) and 8.64 log CFU/ml after 360 min.

During in vitro digestion, the reduction in LAB viability in the gastric phase is generally associated with stress induced by low pH, which compromises cellular integrity. However, several mechanisms contribute to acid resistance in these bacteria, including central metabolic pathways, proton pumps, alterations in membrane composition and cell density, DNA and protein repair systems, neutralization processes, and the expression of stress-related proteins. Together, these mechanisms maintain intracellular homeostasis and restore damaged macromolecules, thereby enabling the recovery of viability during the intestinal phase [38].

During the simulated intestinal phase, characterized by a more neutral to slightly alkaline pH and the presence of bile salts and pancreatic enzymes, a recovery in viable cell counts may occur. This increase should not be attributed exclusively to the resuscitation of “viable but non-culturable” (VBNC) cells, as it may also result from the repair and recovery of sublethally injured cells or limited growth of surviving culturable cells under the less acidic conditions of the intestinal phase. Nevertheless, the resuscitation of VBNC cells represents a plausible mechanism, as LAB have been reported to enter the VBNC state under stressful conditions and subsequently recover cultivability under more favorable environmental conditions. In addition, the food matrix may provide nutrients and protective components that facilitate cellular recovery [39]. Therefore, the increase in viable counts observed during the intestinal phase may reflect a combination of cellular repair, recovery of stressed cells, resuscitation of VBNC cells, and, where conditions permit, growth of surviving cells [40].

Cereals are important sources of dietary fiber, which can be classified according to their solubility in water. Soluble fractions include β-glucans, water-extractable arabinoxylans, arabinoxylooligosaccharides, galactosaccharides, and fructooligosaccharides, whereas insoluble fractions comprise non-extractable arabinoxylans, resistant starch, cellulose, and lignin. Among these, soluble fibers have received considerable attention for their potential use as prebiotic ingredients [41]. Fructooligosaccharides (FOS), for example, are non-digestible carbohydrates commonly used as prebiotics that resist hydrolysis by digestive juices, remaining intact until they reach the intestine.

Previous studies have shown that reducing sugars present in wheat flour can serve as substrates for LAB proliferation during fermentation, contributing to the recovery of viability of certain strains during the intestinal phase [42]. Similarly, millet, owing to its fiber content, complex polysaccharides, and phenolic compounds, can act as a fermentable substrate for LAB, promoting population growth and demonstrating a prebiotic effect [43]. In addition, Zhang et al. [44] demonstrated in animal models that foxtail millet favored the growth of *Lactobacillus*.

The study was primarily designed to compare LAB survival in different flour-based matrices. Future studies using a standardized matrix and different strains or probiotic doses may provide clearer insights into strain-specific performance.

## Conclusion

Functional biscuits intended for healthy young adult dogs were developed using wheat flour, millet flour, and their combination, incorporating the potentially probiotic strains *Lbs. paracasei* GV17 and *L. lactis* GV103, either individually or in combination. The formulations exhibited appropriate color, texture, and proximate composition. LAB counts remained stable in selected formulations over 28 days of refrigerated storage, with the highest survival rates observed in the WF103, MF103, and MF17.103 formulations. Moreover, all formulations demonstrated LAB survival levels exceeding the recommended daily intake for probiotic or functional foods after exposure to simulated GIT conditions. The results confirm that the proposed objective was achieved and highlight the potential of the developed biscuits as effective vehicles for the delivery of potentially probiotic LAB to dogs.

## Supplementary Information

Below is the link to the electronic supplementary material.

Supplementary Material 1

Supplementary Material 2

## Data Availability

The datasets generated during and/or analyzed during the current study are available from the corresponding author on reasonable request.
