# Correction: Lactic acid bacteria, potentially probiotic, in whole-grain pet biscuits: survival and technological potential of millet-based formulations (*Pennisetum glaucum*)

**DOI:** 10.1007/s42770-026-02113-5

**Published:** 2026-10-16

**Authors:** Wendy Carla Dantas Rocha, Isabel Luísa Ribeiro de Abreu Teixeira, Maria Eduarda Costa Campos, Taynan Jonatha Neves Costa, Isabella Maciel Costa, Debora Cristina Sampaio de Assis, Felipe Machado Trombete, Washington Azevedo da Silva, Erika Ramos de Alvarenga, Fabiola de Oliveira Paes Leme, Verônica Ortiz Alvarenga, Bruna Maria Salotti de Souza

**Affiliations:** 1 Department of Technology and Inspection of Products of Animal Origin, School of Veterinary, Federal University of Minas Gerais, Belo Horizonte, 31270-901 Minas Gerais Brazil; 2 Department of Food, Faculty of Pharmacy, Federal University of Minas Gerais, Belo Horizonte, 31270-901 Minas Gerais Brazil; 3 Food Engineering, Federal University of São Joao del Rei, Sete Lagoas, 35702-031 Minas Gerais Brazil; 4 Department of Animal Science, School of Veterinary, Federal University of Minas Gerais, Belo Horizonte, 31270-901 Brazil; 5 Department of Veterinary Clinical and Surgical Medicine, School of Veterinary, Federal University of Minas Gerais, Belo Horizonte, 31270-901 Minas Gerais Brazil; 6 Food Microbiology Laboratory, School of Veterinary, Federal University of Minas Gerais, Belo Horizonte, Brazil

**Correction: Brazilian Journal of Microbiology (2026) 57:266**

https://doi.org/10.1007/s42770-026-02091-8.

The original online version of this article was revised:

Fig. 2 was missing from this article; the figure should have appeared as shown below.

**Figure:**
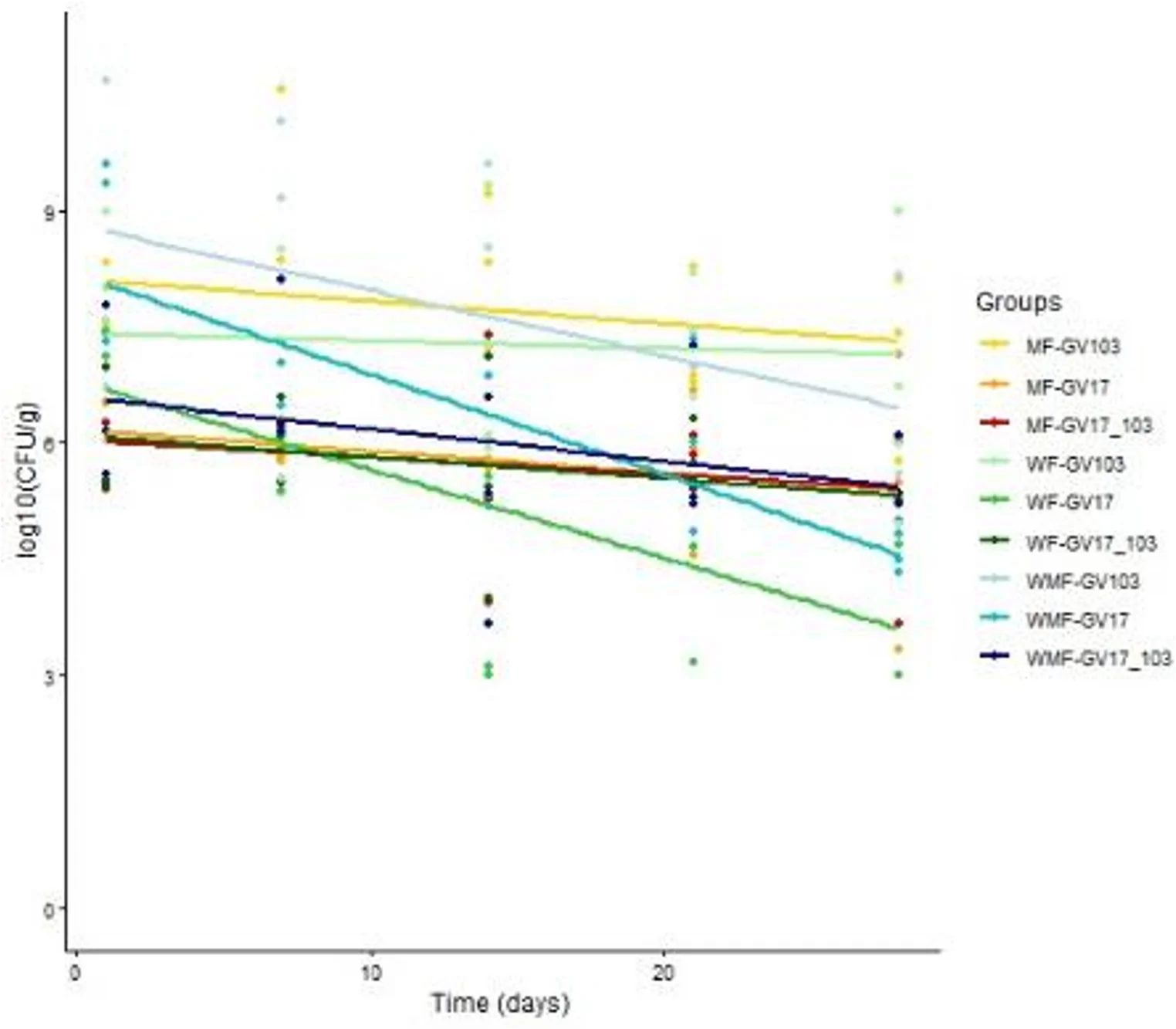

The original article has been corrected.

