# Supplementary material for: Lactic acid bacteria, potentially probiotic, in whole-grain pet biscuits: survival and technological potential of millet-based formulations (Pennisetum glaucum)
Source: Braz J Microbiol. 2026 Sep 10;57(1):266. doi: 10.1007/s42770-026-02091-8 (PMC13562461; doi:10.1007/s42770-026-02091-8)
Supplement: Supplementary file 1 [file 42770_2026_2091_MOESM1_ESM.docx]

**Whole-grain pet biscuits made with millet flour (*Pennisetum glaucum*) and probiotic lactic acid bacteria**

**Supplementary material 2**

**Supplementary material 2.** Regression model of log (bacteria count) over time with different formulation

| **Variable** | **Coefficient** | | **Standard error** | **I.C – 95%** | **p-value** |
| --- | --- | --- | --- | --- | --- |
| Intercept | 7.936 | 0.343 | | [7.26; 8.61] | **<0.0001** |
| GV103 (Reference group) |  |  | |  |  |
| GV17 | -1.768 | 0.247 | | [-2.25; -1.28] | **<0.0001** |
| GV17.103 | -1.726 | 0.247 | | [-2.21; -1.24] | **<0.0001** |
| MF (Reference group) |  |  | |  |  |
| WF | -0.003 | 0.44 | | [-0.87; 0.86] | 0.994 |
| WMF | 1.093 | 0.44 | | [0.23; 1.96] | **0.014** |
| Time | -0.026 | 0.018 | | [-0.06; 0.01] | 0.151 |
| WF:Time | -0.024 | 0.026 | | [-0.07; 0.03] | 0.346 |
| WMF:Time | -0.059 | 0.026 | | [-0.11; -0.01] | **0.024** |

GV17: *Lbs. paracasei* GV17; GV17.103: association of *Lbs. paracasei* GV17 and *L. lactis* GV103; WF: wheat flour; WMF: wheat and millet flour. Bacteria lineage and formulation were coded as indicator variables (binary variables) and GV103 and MF were the reference groups for these variables, respectively. R^2^ = 0.451, Adjusted R^2^ = 0.42 and maximum R^2^ of 0.77.
