# Supplementary material for: Lactic acid bacteria, potentially probiotic, in whole-grain pet biscuits: survival and technological potential of millet-based formulations (Pennisetum glaucum)
Source: Braz J Microbiol. 2026 Sep 10;57(1):266. doi: 10.1007/s42770-026-02091-8 (PMC13562461; doi:10.1007/s42770-026-02091-8)
Supplement: Supplementary file 2 [file 42770_2026_2091_MOESM2_ESM.docx]

**Whole-grain pet biscuits made with millet flour (*Pennisetum glaucum*) and probiotic lactic acid bacteria**

**Supplementary material 1**

**Supplementary material 1 1.** Dog biscuits made with wheat flour - WF (A), millet flour - MF (B), and both wheat and millet flour – WMF (C)
